# Poster Session I – A89 FOOD FOR MOOD: COMPARING THE EFFECTS OF THE MEDITERRANEAN DIET AND WESTERN DIET ON MENTAL HEALTH

**DOI:** 10.1093/jcag/gwaf042.089

**Published:** 2026-02-13

**Authors:** A Lewis, N Haskey, M Raman

**Affiliations:** Cumming School of Medicine, University of Calgary, Calgary, AB, Canada; The University of British Columbia, Kelowna, BC, Canada; Cumming School of Medicine, University of Calgary, Calgary, AB, Canada

## Abstract

**Background:**

Anxiety and depression (A/D) affect 5% of the global population, yet only one in five receives adequate management. Problematic, as co-morbid A/D is common in chronic digestive diseases and negatively influences prognosis and management. Cross-sectional studies associate poor diet quality and high intake of ultra-processed foods (UPFs) with an increased risk of A/D, while adherence to a Mediterranean-style diet (MD) has been linked to improvements in A/D severity. Their comparative effects on mental health outcomes in a controlled feeding trial are unknown.

**Aims:**

To determine whether adherence to a MD improves symptoms of A/D compared with a UPF diet, using a controlled feeding design.

**Methods:**

Healthy adults (n = 18) completed a randomized controlled feeding, crossover trial. Eligibility criteria included absence of acute or chronic physical or mental health conditions, medication or substance use, or prior major surgery. Participants consumed a fully catered 3-week UPF diet (85.5% NOVA 4), followed by a 6-week washout period, then a 3-week MD (8.9% NOVA 4). Each diet provided three meals and two snacks daily, matched in energy and macronutrient quantity. The Generalized Anxiety Disorder 7-item (GAD-7) scale, the Patient Health Questionnaire 8-item (PHQ-8), and the Short Form-12 Health Survey (SF-12) questionnaire were administered at baseline and at the end of week 3 of each diet arm. Statistical analysis was performed using the Mann-Whitney U and the Wilcoxon matched-pairs signed rank test.

**Results:**

Eighteen participants (50% female) completed the study, with a median age of 24.5 years [IQR: 23.0-29.0], and a median Body Mass Index of 24.3 kg/m^2^ [IQR: 23.2, 25.2]. The Healthy Eating Index (HEI) score was significantly higher in MD compared to the UPF (MD: 83.7 [IQR: 82.5-87.50] WD: 41.1 [IQR: 36.4-49.6], p < 0.0001).

PHQ-8 scores increased following the UPF diet (median +3.5, p = 0.002), reaching a median score consistent with mild depressive symptoms. At week 3, PHQ-8 scores were significantly higher in the UPF group compared to the MD (median 3.5-point difference, p = 0.004). Quality of life measures declined during the UPF diet, significantly reducing the Mental Component Score (MCS: -8.3%, p = 0.003) and Physical Component Score (PCS: -4.1% p = 0.004). Between-group comparisons at week 3 demonstrated that MCS and PCS improved during the MD compared to the UPF diet (MCS: +6.1, p = 0.009, PCS: +2.4, p = 0.005). There were no significant differences in GAD-7 scores.

**Conclusions:**

Short-term adherence to a UPF diet worsened depressive symptoms and reduced quality of life, while the MD improved or maintained these outcomes. These findings underscore the rapid influence of diet quality on mental well-being and support further research into diet as an adjunct treatment for chronic digestive diseases.

**Funding Agencies:**

Weston Family Foundation

